# Biomarker Phenotype for Early Diagnosis and Triage of Sepsis to the Pediatric Intensive Care Unit

**DOI:** 10.1038/s41598-018-35000-7

**Published:** 2018-11-09

**Authors:** Beata Mickiewicz, Graham C. Thompson, Jaime Blackwood, Craig N. Jenne, Brent W. Winston, Hans J. Vogel, Ari R. Joffe

**Affiliations:** 1 Bio-NMR-Centre, Department of Biological Sciences, Faculty of Science, University of Calgary, Calgary, AB Canada; 2 Department of Pediatrics, University of Calgary, Calgary, AB Canada; 3 Department of Microbiology, Immunology and Infectious Diseases, Cumming School of Medicine, University of Calgary, Calgary, AB Canada; 4 Department of Critical Care Medicine, Department of Medicine, Department of Biochemistry and Molecular Biology, Cumming School of Medicine, University of Calgary, Calgary, AB Canada; 5 Division of Pediatric Critical Care Medicine, Department of Pediatrics, University of Alberta, Edmonton, AB Canada

## Abstract

Early diagnosis and triage of sepsis improves outcomes. We aimed to identify biomarkers that may advance diagnosis and triage of pediatric sepsis. Serum and plasma samples were collected from young children (1–23 months old) with sepsis on presentation to the Pediatric Intensive Care Unit (PICU-sepsis, n = 46) or Pediatric Emergency Department (PED-sepsis, n = 58) and PED-non-sepsis patients (n = 19). Multivariate analysis was applied to distinguish between patient groups. Results were compared to our results for older children (2–17 years old). Common metabolites and protein-mediators were validated as potential biomarkers for a sepsis-triage model to differentiate PICU-sepsis from PED-sepsis in children age 1 month-17 years. Metabolomics in young children clearly separated the PICU-sepsis and PED-sepsis cohorts: sensitivity 0.71, specificity 0.93, and AUROC = 0.90 ± 0.03. Adding protein-mediators to the model did not improve performance. The seven metabolites common to the young and older children were used to create the sepsis-triage model. Validation of the sepsis-triage model resulted in sensitivity: 0.83 ± 0.02, specificity: 0.88 ± 0.05 and AUROC 0.93 ± 0.02. The metabolic-based biomarkers predicted which sepsis patients required care in a PICU versus those that could be safely cared for outside of a PICU. This has potential to inform appropriate triage of pediatric sepsis, particularly in EDs with less experience evaluating children.

## Introduction

Sepsis is defined as life-threating organ dysfunction caused by a dysregulated host response to infection^1^. If the diagnosis of sepsis is missed due to nonspecific presentations, sepsis may deteriorate to septic shock in which underlying circulatory and cellular/metabolic abnormalities substantially increase patient mortality^2^. It has been estimated that infections account for almost 60% of all deaths in children younger than 5 years old^3^. In the U.S.A. sepsis causes over 75,000 hospitalizations and nearly 10,000 deaths every year in children^4^. Moreover, the prevalence of sepsis and sepsis-associated mortality have been significantly higher in neonates and infants than in any other subgroup of pediatric (age < 20 years) patients^4,5^. Despite advances in methods for detecting microbial pathogens, the underlying cause of pediatric sepsis cannot be clearly identified in over 75% of cases^6^. The proper diagnosis of sepsis remains a priority for the health care system; thus, it is necessary to develop new techniques and identify reliable biomarkers to improve sepsis diagnosis and prognosis. Early diagnosis of sepsis followed by correct triage of patients (i.e. patient admission to a hospital ward for observation or to a pediatric intensive care unit (PICU)) and initiation of appropriate treatment are associated with improved patient outcome^7,8^. Frequently, these initial important decisions are left to physician judgement^7,9^. Physician choices may significantly differ between pediatric emergency departments (PED) with extensive experience with sepsis in children and smaller centers without such knowledge^10,11^.

In our previous study we found that in children 2–17 years old, who are likely to have a mature immune system, metabolic and inflammatory protein-mediator profiling could be utilized to differentiate children with sepsis requiring care in a PICU from children with and without sepsis safely cared for outside of a PICU^12^. Integrating these two sources of biomarker data may improve prediction by increasing the information available describing the state of the organism. This systems biology-based approach may realistically lead to development of a point-of-care test aiding sepsis triage decisions, but in addition needs to be examined in a younger pediatric cohort (under 2 years old) who may have a less mature or developing immune response to sepsis. Therefore, here we have investigated whether metabolomics and inflammatory protein-mediator profiling can be applied for early diagnosis and triage of sepsis in infants (1–23 months old). Next, we selected the most significant metabolites and inflammatory protein-mediators responsible for sepsis triage and we validated these potential biomarkers in the entire pediatric cohort (children age 1 month-17 years). Specifically, we hypothesized that a biopattern can be identified that differentiates between children with sepsis that require care in a PICU versus those that can be safely cared for outside of a PICU.

## Methods

### Patients and sample collection

The study was approved by the Conjoint Health Research Ethics Board of the University of Calgary (Ethics ID 23426), and the Health Research Ethics Board of the University of Alberta (Pro00008797), and all patients, or their next of kin, provided written informed consent for participating in this study. All samples were collected in accordance with the Tri-Council policy statement. The sample collection protocol has previously been described in detail^12^. Briefly, samples were obtained from an existing arterial or central venous catheter (PICU-sepsis cohort) or with intravenous insertion or blood culture draw (PED-cohorts). Blood was drawn as soon as possible (within 24 hours of meeting eligibility).

#### PICU-sepsis cohort

All eligible children admitted to the only two PICUs in Alberta with a diagnosis of sepsis were prospectively enrolled (04/2010–10/2013). Sepsis was defined as systemic inflammatory response syndrome (SIRS), caused by a suspected/proven bacterial/fungal infection, with antibiotics ordered, and an arterial and/or central venous line in place. Patients were excluded if: expected survival ≤24 h, decision for palliation, or having severe sepsis for ≥48 h (in order to exclude those with late presentation of severe-sepsis to the PICU). Patients with microbiologically confirmed sepsis (positive culture from a normally sterile site, including blood, cerebrospinal fluid, peritoneal fluid, or tissue) or pneumonia without microbiological confirmation (SIRS with chest infiltrate suggesting pneumonia) had specimens analyzed. Site of infection was defined as that diagnosed by the attending medical team. Blood was drawn as soon as possible upon PICU admission using a deferred consent model, except for the few patients that had blood drawn in the PED (2/46 (4%) and 3/94 (3%) of the PICU-sepsis cohort age 1–23 months and 2–17 years respectively).

#### PED-sepsis cohort

Children admitted to one PED in Alberta with a diagnosis of sepsis were prospectively enrolled. Sepsis was defined as SIRS caused by a suspected or proven bacterial or fungal infection, with antibiotics and blood culture ordered. If a patient was admitted to the PICU from the PED, they were only included in the PICU-sepsis cohort. The definition of PICU-sepsis was more stringent (excluding those without clear bacterial sepsis, e.g., with unknown, viral, or other causes of systemic inflammatory response syndrome) than in the PED-sepsis cohort. This was for both operational reasons (inability to follow the PED-sepsis cohort throughout their hospital [n = 35] or outpatient management), and to identify the PICU-sepsis cohort as those with serious infection requiring care in a PICU.

#### PED-control cohort

In the same PED, previously healthy children were prospectively enrolled if they were admitted for a procedure requiring intravenous sedation/analgesia, and without an infection (no history of fever within 2 weeks, and no clinical evidence of infection).

### Sample preparation and data collection

#### ^1^H Nuclear magnetic resonance (NMR) spectroscopy

The sample preparation protocol and NMR spectral acquisition are presented in the Supplementary Methods and have previously been described in detail^12–14^. Briefly, high resolution one-dimensional ^1^H NMR spectra of filtered serum samples were obtained on a 600 MHz Bruker Ultrashield Plus NMR spectrometer (Bruker BioSpin Ltd., Canada). The spectra were profiled in Chenomx NMR Suite 7.5 software (Chenomx Inc., Edmonton, AB, Canada)^15^. The concentration of internal standard (0.5 mM) was used to determine the concentrations of detected metabolites. All samples and ^1^H NMR spectra were randomized prior to analysis to avoid progressive bias.

#### Inflammatory protein-mediators

Quantification of targeted inflammatory protein-mediators (cytokines, chemokines and acute-phase proteins that are involved in inflammation) was done using validated Luminex bead-based multiplexing assays according to the manufacturer’s instructions, as described previously^12,16^.

### Statistical analysis

For detailed information about data pre-processing and statistical data analysis see the Supplementary Methods. Briefly, multivariate statistical analysis was performed using SIMCA-P+ 12.0.1 software (Umetrics, Sweden) and consisted of principal component analysis (PCA) and orthogonal partial least squares discriminant analysis (OPLS-DA). For each OPLS-DA model the R2Y (the percentage of variation explained by the model), Q2 (the predictive ability of the model) and CV-ANOVA (Cross-Validated Analysis Of Variance) p-value^17,18^ were calculated based on sevenfold cross-validation (CV)^19^. The significant metabolites and protein-mediators were selected from the OPLS-DA regression coefficients (p < 0.05; jackknife technique)^20^.

To confirm potential biomarkers for sepsis triage the results for infants were compared to our previously published results for older children (2–17 years old)^12^. Based on the common *biopattern* for both pediatric cohorts alternative OPLS-DA models were created for each age group and for the combined group. The receiver operating characteristic (ROC) analysis^21^ was performed for each single potential biomarker, and for the multivariate OPLS-DA model biopatterns. To validate the sepsis triage model based on the common potential biomarkers, the OPLS-DA model which consisted of 2/3 of all available pediatric samples was used as a training set and the remaining samples comprised a test set. The model was validated 3 times and for each training set the samples were randomly selected.

## Results

### Patient cohort

The demographics, sites of infection, and severity-of-illness measures for each cohort are provided in Table 1. Eligible children were prospectively registered into the database after deferred consent was obtained; the consent rate was 78%. Of 63 patients age <24 months, those without clear bacterial sepsis (unknown, viral, or other causes of *systemic inflammatory response syndrome*) were excluded from the PICU-sepsis nested cohort. Most (>90%) of the PICU-sepsis cohort developed sepsis and were enrolled on the day of PICU admission (median PICU length of stay at enrollment 0 [IQR 1.0] days; 4 (8.7%) enrolled after day 1 of PICU stay; Table 1), with the large majority (>90%) admitted directly from an ED to PICU (only 2 (4%) had study labwork sent while in the PED)^22^. The first lactate on the day of enrolment was 1.9 (SD 1.4) mmol/L, suggesting early enrolment. On the day of enrolment, the PICU-sepsis cohort patients (n = 46) had PEdiatric Logistic Organ Dysfunction scores and Pediatric RISk of Mortality III scores comparable to other PICU sepsis trial patients^23,24^. Most (90%) PICU-sepsis patients were ventilated (for a median of 8 days) during the index sepsis episode, and 50% received vasoactive infusions on the first day of sepsis. The median PICU length of stay after sepsis was 10 days, with only 4% staying in PICU <2 days after enrolment (Table 1). There were no statistically significant differences between the two hospital’s PICU-sepsis cohorts in day of enrolment mechanical ventilation, WBC count, lactate, PRISM score, or PELOD score, or in the duration of ventilation or PICU stay after inclusion [data not shown]. In addition, we found no statistically significant difference between the two PICU-sepsis cohorts in a combined metabolic and protein-mediator model [data not shown]. The PED-sepsis cohort showed comparable demographics, except for having fewer comorbidities and better values for platelet count and systolic blood pressure. No patient in the PED-sepsis cohort was admitted to a PICU within 7 days of enrolment.

**Table 1:** Description of the pediatric patient cohorts.

Descriptive variable	PICU-sepsis cohort	PED-sepsis cohort	PED-control cohort
Number of patients	n = 45^a^	n = 58	n = 19
Age (months)	9.8 (7.4)	11.2 (6.7)	11.7 (9.5)
Gender: male	28 (61%)	31 (53%)	11 (58%)
Weight (kg)	8.1 (3.2)	9.1 (2.6)	11.1 (1.6)
Underlying co-morbidity			0 (0%)
-Neuromuscular	6 (13%)	1 (2%)	
-Cardiac	10 (22%)	1 (2%)	
-Respiratory	9 (20%)	1 (2%)	
-Chromosomal abnormality	3 (7%)	2 (3%)	
PRISM III score	8.6 (6.8); 6 [3,14]	N/A	N/A
PELOD score	15.0 (9.0); 12 [11,21]	N/A	N/A
WBC	12.6 (7.4)	12.3 (7.2); n = 55	N/A
Platelet count	239 (161)	314 (132); n = 54	N/A
Creatinine (μmol/L)	30 (19)	26 (21); n = 45	N/A
Lactate (mmol/L)	1.9 (1.4)	1.7 (0.4);n = 9	N/A
Lowest SBP (mmHg)	76 (14)	95 (14)	110 (13)
Lowest MAP (mmHg)	52 (10)	—	—
pH	7.3 (0.1)	7.39 (0.13); n = 13	—
Sepsis developed after first PICU day	4 (9%)	N/A	N/A
Site of infection			N/A^d^
-Pneumonia without microbiological confirmation	21 (46%)	11 (19%)	
-Microbiologically confirmed (culture positive)	23 (50%)^b^	21 (36%)^c^	
-Clinically diagnosed	0 (0%)	26 (45%)^c^	
Mechanical ventilation on first day	35 (76%)	N/A	N/A
Inotrope/vasopressor infusion on first day	23 (50%)	N/A	N/A
Duration of mechanical ventilation after enrolment (days)	n = 40 (90%); 8 [4, 11]	N/A	N/A
PICU length of stay after enrolment (days)	<2 days: 2 (4%) 10 [6, 14]	Hospital stay 4 [3, 5]; n = 35	N/A
RRT	3 (7%)	0 (0%)	N/A
ECLS therapy	2 (4%)	0 (0%)	N/A
PICU Mortality	2 (4%)	0 (0%)	0 (0%)

Results given as n (%) or mean (SD) or median [IQR].

ECLS: extracorporeal life support; MAP: mean arterial pressure; N/A: not applicable; PED: pediatric emergency department; PELOD: pediatric logistic organ dysfunction score; PICU: pediatric intensive care unit; PRISM: pediatric risk of mortality score; RRT: renal replacement therapy; SBP: systolic blood pressure.

^a^One patient withdrew consent to use their demographic and clinical data.

^b^Sites of infection included: meningitis (9; 3 with bacteremia); empyema (1); bacteremia (8; 1 with pneumonia, 2 from central venous line source); peritonitis (2; both from gut ischemia); fasciitis (1, of abdominal wall); mediastinitis (1), parapharyngeal abscess from Streptococcus pyogenes (1).

^c^Sites of infection included: a. Microbiologically confirmed- meningitis (2, both with bacteremia); bacteremia (9; 2 with pneumonia, 1 with UTI, 1 with cervical adenitis); urinary tract infection (11); MRSA skin abscess (1). b. Clinically diagnosed: retropharyngeal cellulitis (2), otitis media (1), cervical adenitis (1), neutropenia with fever (1), presumed viral febrile illness (8; 4 confirmed as RSV-2, Influenza-1, HSV stomatitis-1), and unknown source of fever (13).

^d^Procedures for: orthopedic reduction 4; laceration repair 10; foreign body removal 1; other 4 (amputation revision 1, casting 2, plastibell removal from penis 1).

### Metabolic profiling dataset

A total of 59 metabolites were detected, assigned and quantified in each serum sample. Acetaminophen and propylene glycol were excluded from the statistical analysis as they are related to medication and treatment^25^, and glycerol, a potential contaminant from the microcentrifuge filters, was also excluded. Additionally, the ethanol concentration could not be determined in more than half of samples as its metabolite peaks were in the noise level in the NMR spectra (i.e., below the threshold for reliable detection). Consequently, the ^1^H NMR dataset used for statistical analysis consisted of 55 metabolites (Supplementary Table S1) and 3.1% of missing values which were randomly distributed.

**Figure 1:**
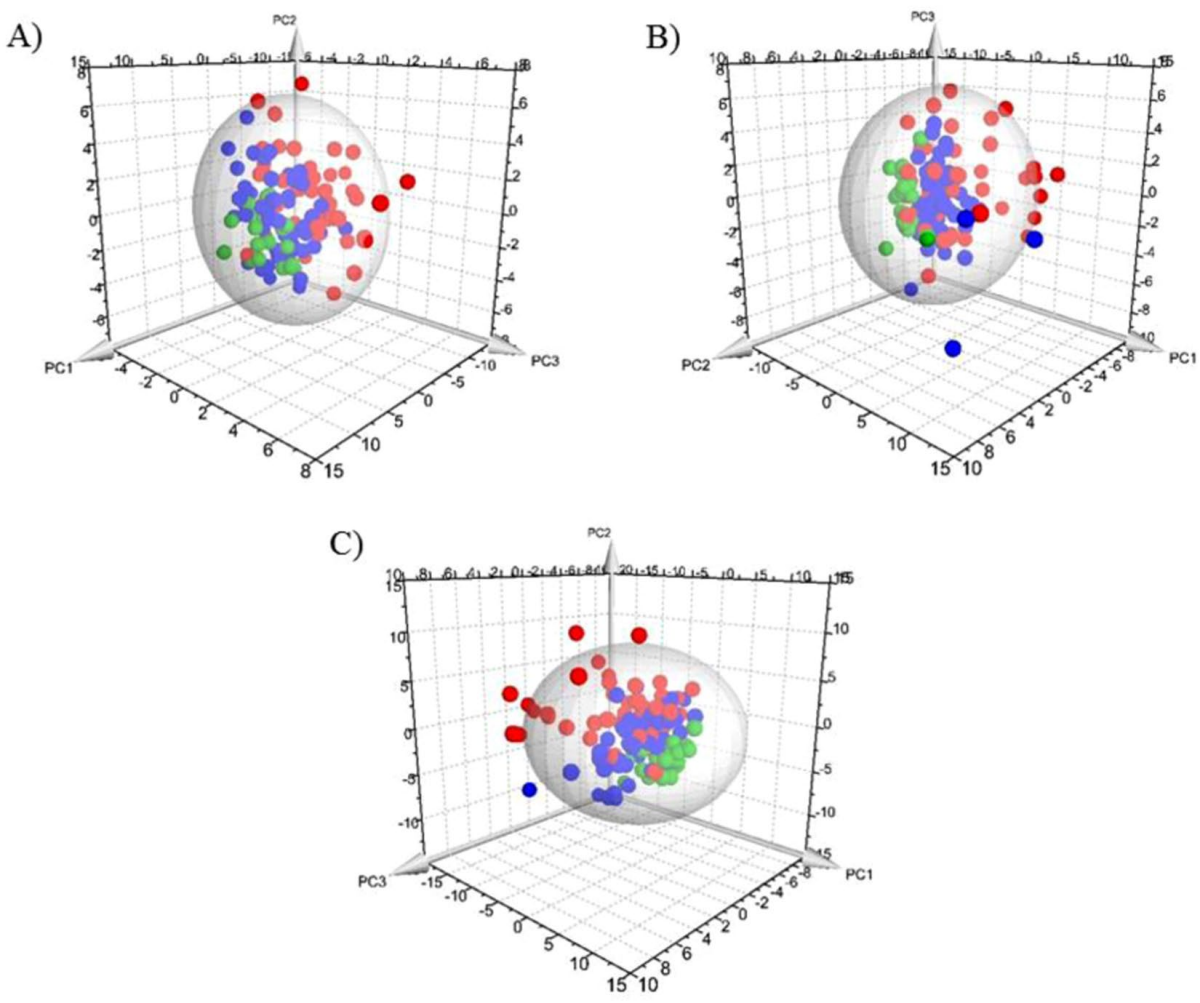
Principal Component Analysis results for the 1–23 month old cohorts. (**A**) Metabolic data profiling, (**B**) inflammatory protein-mediator data profiling, and (**C**) combined biomarker profiling data. Red dots: PICU-sepsis patients; blue dots: PED-sepsis cohort; green dots: PED-control. The sphere describes the 95% confidence interval of the Hotelling’s T-squared distribution.

The PCA model was based on three principal components which in total summarized 36.2% of variation in the dataset (Fig. 1A). Four PICU-sepsis samples were detected as outliers (1/8 (13%) of the venous samples, and 3/38 (8%) of the arterial samples) and were further excluded from analyses; outliers can potentially bias the results of supervised analysis^18^.

**Figure 2:**
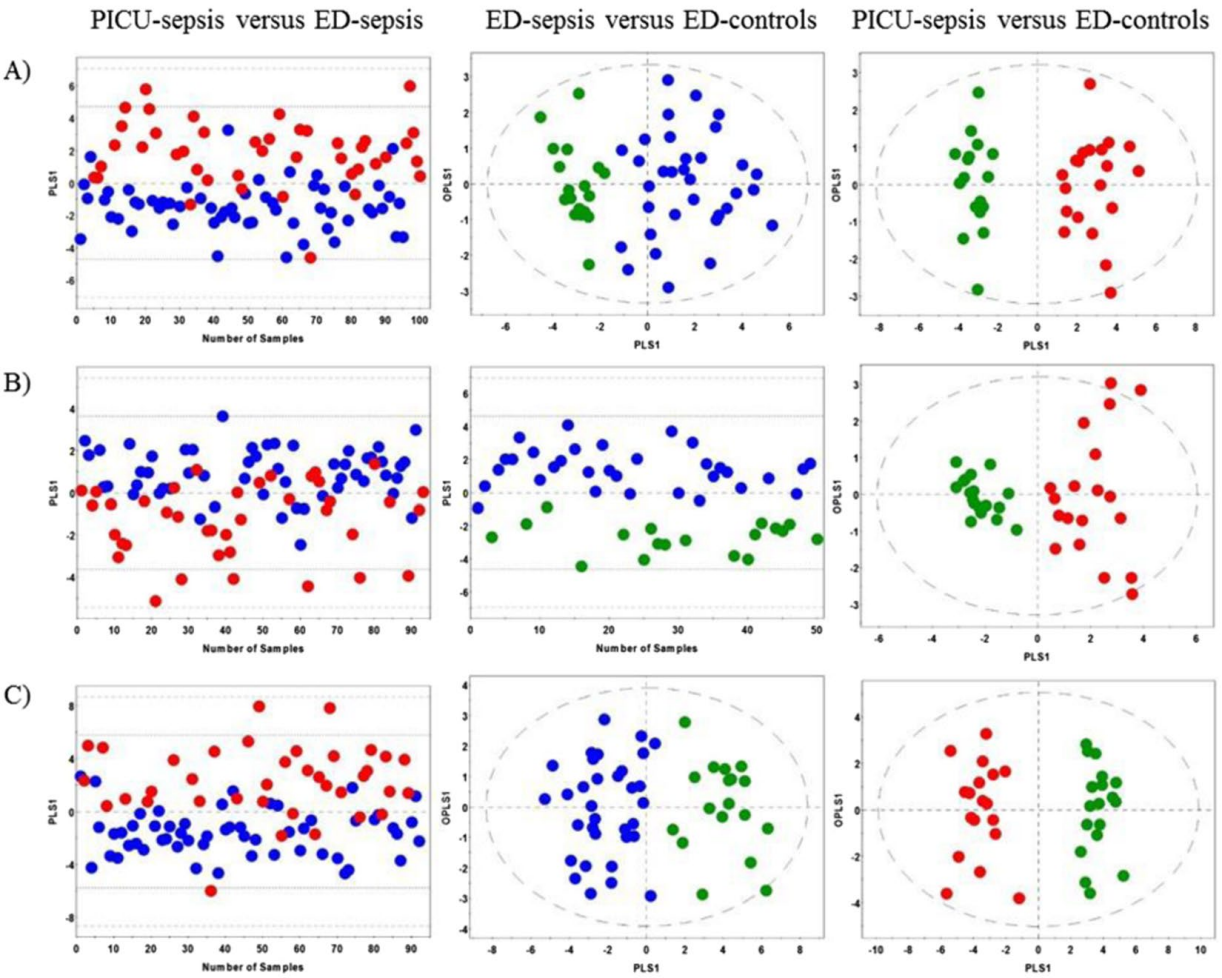
Orthogonal Partial Least Squares Discriminant Analysis for the 1–23 month old cohorts. (**A**) Metabolic data profiling, (**B**) inflammatory protein-mediator data profiling, and (**C**) combined biomarker profiling data. Red dots: PICU-sepsis cohort; blue dots: PED-sepsis cohort; green dots: PED-control cohort.

To better reveal metabolic differences between two specific patient cohorts OPLS-DA analysis was carried out (Fig. 2A). The OPLS-DA score scatter plots demonstrated clear separation between patient cohorts with high values of R2Y and Q2 metrics and significant CV-ANOAVA p-values (Fig. 2A and Table 2). The ROC analysis (Table 2) revealed high AUROC values: AUROC = 0.90 ± 0.03 for PICU-sepsis versus PED-sepsis, AUROC = 0.99 ± 0.01 for PED-sepsis versus PED-controls and AUROC = 1.0 for PICU-sepsis versus PED-controls.

**Table 2:** Results of orthogonal partial least squares discriminant analysis for the 1–23 month old cohorts.

Patient cohort	Data	R2Y	Q2	CV-ANOVA p-value	Sensitivity: Specificity	PPV:NPV	Accuracy	AUROC ± standard error
PICU-sepsis versus PED-sepsis	Metabolites	0.48	0.44	8.5 × 10^−13^	0.71:0.93	0.88:0.82	0.84	0.90 ± 0.03
	Mediators	0.37	0.32	3.0 × 10^−8^	0.56:0.87	0.76:0.73	0.74	0.84 ± 0.04
	Combined	0.45	0.39	2.5 × 10^−10^	0.70:0.91	0.84:0.82	0.83	0.87 ± 0.04
PED-sepsis versus PED-controls	Metabolites	0.72	0.61	7.8 × 10^−9^	0.91:1.0	1.0:0.86	0.94	0.99 ± 0.01
	Mediators	0.77	0.75	1.2 × 10^−14^	0.97:0.94	0.97:0.94	0.96	0.99 ± 0.003
	Combined	0.80	0.70	1.6 × 10^−11^	1.0:0.94	0.97:1.0	0.98	0.99 ± 0.02
PICU-sepsis versus PED-controls	Metabolites	0.93	0.88	1.9 × 10^−14^	1.0:1.0	1.0:1.0	1.0	1.0
	Mediators	0.86	0.67	2.7 × 10^−7^	0.89:0.94	0.94:0.89	0.92	0.99 ± 0.01
	Combined	0.94	0.90	7.6 × 10^−15^	1.0:1.0	1.0:1.0	1.0	1.0

AUROC: area under the receiver operating characteristic curve; PICU: pediatric intensive care unit; PED: emergency department; R2Y: percentage of variation explained by the model; Q2: predictive ability of the model; CV-ANOVA: cross-validated analysis of variance; PPV:NPV: positive predictive value: negative predictive value.

### Inflammatory protein-mediator profiling dataset

Overall, 57 inflammatory protein-mediators were recognized and quantified in the plasma samples (Supplementary Table S2). Fibrinogen, serum amyloid A, haptoglobin and C-reactive protein were excluded from the statistical analysis as they were showing “out-of-range” values (above upper limit of detection) for more than 50% of the samples. The final dataset consisted of 53 protein-mediators and 3.1% of missing values (randomly distributed).

Three principal components were calculated for the PCA model and the total variation summarized by the model was 46.7% (Fig. 1B). Eleven samples were detected as outliers: 7 PICU-sepsis samples and 4 PED-sepsis samples. Similar to the ^1^H NMR dataset these outliers were excluded in further statistical models as is recommended for this type of analysis^18^.

The OPLS-DA models for the PED-sepsis cohort versus PED-controls and the PICU-sepsis cohort versus PED-controls demonstrated very good separation between the patient groups with high values of validation parameters and significant CV-ANOVA p-values (Fig. 2B and Table 2). However, the OPLS-DA model for the PICU-sepsis cohort versus PED-sepsis patients had lower values of R2Y, Q2 and AUROC parameters when compared to the other OPLS-DA models: R2Y = 0.37, Q2 = 0.32, AUROC = 0.84 ± 0.04 (Fig. 2B and Table 2).

### Combined metabolic and protein-mediator profiling dataset

Three principal components explained 38.3% of the total variation in the combined metabolic and inflammatory protein-mediators profiling dataset (Fig. 1C). There were 12 outliers: 9 PICU-sepsis samples and 3 PED-sepsis samples. All outlying samples were excluded in subsequent supervised analyses. Of note, only 1 patient was an outlier in all three analyses; on review there were no obvious clinical reasons for this.

The OPLS-DA score scatter plots clearly distinguished between the patient groups and were associated with high values of R2Y and Q2 parameters, and significant CV-ANOVA p-values (Fig. 2C and Table 2). The most powerful combined metabolites/protein-mediators OPLS-DA model was constructed for the PICU-sepsis patients versus the PED-controls: R2Y = 0.94, Q2 = 0.90 and AUROC = 1.0. Interestingly, the PICU-sepsis versus PED-sepsis OPLS-DA model for the ^1^H NMR-based data was superior when considering all implemented approaches, while the OPLS-DA model for PED-sepsis versus PED-controls showed very similar results for the metabolomics, protein mediators and combined datasets (Table 2).

### Supervised models without outliers excluded

To confirm that excluding the outliers from the supervised analyses did not bias the results, we recalculated the OPLS-DA models including all outlying samples which were detected in the PCA models. Leaving the outliers out from the supervised analyses only had a minor influence on the discriminative and predictive ability of the models (Supplementary Table S3). Of note, the subsequent combined age 1 month to 17 years old models were analysed without any outliers excluded.

### Potential biomarkers and model validation

Overall, 21 metabolites and 15 inflammatory protein-mediators were responsible for the separation between the PICU-sepsis patients and the PED-sepsis cohort (Supplementary Table S4; Supplementary Fig. S1). After comparing these results to the previously published outcomes of metabolite and protein-mediator profiling for 2–17 year old children (Supplementary Table S5 and Supplementary Fig. S1)^12^ we found that 7 metabolites and their concentration changes were common to infants and older children: increased level of dimethylamine, mannose, 3-methyl-2-oxovalerate and 3-hydroxyisovalerate, and decreased concentrations of alanine, O-acetylcholine and acetate. None of the inflammatory protein-mediators detected in the OPLS-DA model (combined dataset) for infants PICU-sepsis versus PED-sepsis was common to the same type of model for the older children.

Based on these 7 metabolites alternative OPLS-DA models for PICU-sepsis versus PED-sepsis were constructed for infants (n = 104) and older children (n = 175) respectively, without excluding outliers. Both models revealed highly significant values of validation parameters (Table 3). The ROC analysis performed for a single potential biomarker and the 7 metabolites taken together demonstrated that the highest AUROC values were obtained for the multivariate *biopatterns* (Table 4): AUROC = 0.91 ± 0.03 for infants, and AUROC = 0.93 ± 0.02 for the 2–17 year old cohort.

**Table 3:** Results of orthogonal partial least squares discriminant analysis based on 7 potential biomarkers.

PICU-sepsis versus PED-sepsis	R2Y	Q2	CV-ANOVA p-value	Sensitivity: Specificity	PPV:NPV	Accuracy	AUROC ± standard error
1–23 month old cohort	0.46	0.44	1.8 × 10^−13^	0.80:0.88	0.84:0.85	0.85	0.90 ± 0.03
2–17 year old cohort	0.60	0.58	2.7 × 10^−33^	0.87:0.93	0.93:0.86	0.90	0.95 ± 0.02
1 month-17 year old cohort	0.53	0.52	2.3 × 10^−22^	0.84:0.89	0.89:0.84	0.86	0.93 ± 0.02

AUROC: area under the receiver operating characteristic curve; PICU: pediatric intensive care unit; PED: emergency department; R2Y: percentage of variation explained by the model; Q2: predictive ability of the model; CV-ANOVA: cross-validated analysis of variance; PPV:NPV: positive predictive value: negative predictive value.

**Table 4:** Results of receiver operating characteristic analysis for each single potential biomarker and the multivariate *biopattern* for differentiating sepsis requiring care in pediatric intensive care from sepsis cared for in the emergency department.

Potential biomarkers	AUROC ± standard error	
	1–23 month old cohort	2–17 year old cohort
3-Hydroxyisovalerate	0.65 ± 0.05	0.71 ± 0.04
3-Methyl-2-oxovalerate	0.64 ± 0.06	0.62 ± 0.04
Acetate	0.73 ± 0.05	0.86 ± 0.03
Alanine	0.84 ± 0.04	0.75 ± 0.04
Dimethylamine	0.79 ± 0.04	0.77 ± 0.04
Mannose	0.75 ± 0.05	0.80 ± 0.03
O-Acetylcholine	0.76 ± 0.05	0.75 ± 0.04
**Multivariate** ***biopattern***	0.91 ± 0.03	0.93 ± 0.02

Finally, the OPLS-DA model for all pediatric patients (1 month-17 years old, n = 279) PICU-sepsis versus PED-sepsis, which was based on the multivariate *biopattern* (7 potential biomarkers), was created: R2Y = 0.53, Q2 = 0.52, and AUROC = 0.93 ± 0.02 (Table 3). The model was validated 3 times using 186 randomly selected pediatric samples as a training set and the remaining samples (n = 93) as a test set (Table 5). The mean of the predicted response values for PICU-sepsis samples in the test sets was 0.8 ± 0.2 and for PED-sepsis was 0.2 ± 0.1. The average sensitivity, specificity, accuracy and AUROC in these test sets were 0.83 ± 0.02, 0.88 ± 0.05, 0.85 ± 0.01 and 0.93 ± 0.02 respectively.

**Table 5:** Validation results for the orthogonal partial least squares discriminant analysis model for differentiating sepsis requiring care in pediatric intensive care from sepsis cared for in the emergency department based on the multivariate *biopattern* in children age 1 month-17 years.

Validation		Training set (n = 186)	Test set (n = 93)	Predicted response value ± standard deviation	Sensitivity: Specificity	Accuracy	AUROC ± standard error
#1	PICU-sepsis	n = 90	n = 50	0.78 ± 0.27	0.84:0.86	0.85	0.94 ± 0.02
	PED-sepsis	n = 96	n = 43	0.24 ± 0.21			
#2	PICU-sepsis	n = 93	n = 47	0.78 ± 0.34	0.85:0.85	0.85	0.92 ± 0.03
	PED-sepsis	n = 93	n = 46	0.21 ± 0.28			
#3	PICU-sepsis	n = 83	n = 57	0.74 ± 0.27	0.81:0.94	0.86	0.93 ± 0.03
	PED-sepsis	n = 103	n = 36	0.23 ± 0.22			

AUROC: area under the receiver operating characteristic curve; PICU: pediatric intensive care unit; PED: emergency department.

## Discussion

Sepsis remains a significant cause of morbidity and mortality for pediatric patients worldwide^3,5,7^, hence identifying improved diagnostic and prognostic tools remains a priority. Here we have utilized metabolomics and inflammatory protein-mediator profiling approaches to investigate whether these methods and/or their combination could be used for the diagnosis and triage of sepsis in infants. Through multivariate statistical analysis we were able to identify specific *biopatterns* in infants (1–23 months old) associated with early differentiation of PED-sepsis patients from PICU-sepsis patients requiring care in the PICU (Table 2). Moreover, by comparing and integrating the results with our previously published findings for older children we defined and validated potential biomarkers responsible for distinguishing a PICU-sepsis cohort from a PED-sepsis cohort for children 1 month-17 years old. We have identified 7 metabolites that can be described as a *biopattern* for triage of sepsis in children (Table 4).

Metabolomics examines the end result of interactions among the environment and gene expression on metabolism in the organism; as such, if levels of certain metabolites are either increased or decreased, this reflects modulation of the pathways involved in producing those metabolites. The pediatric sepsis triage model based on the 7 potential biomarkers and their concentration changes was submitted for topological metabolic pathway analysis in MetaboAnalyst 3.0 software^26^. This revealed a number of impacted metabolic pathways (Table 6) within PICU-sepsis and PED-sepsis cohorts: energy metabolism (e.g. pyruvate metabolism) and amino acid metabolism (e.g. alanine, aspartate and glutamate metabolism; valine, leucine and isoleucine degradation) were the most perturbed pathways. Adult studies have reported similar impacted metabolic pathways in sepsis, including amino acid metabolism, and mitochondrial energy metabolism^27^.

**Table 6:** The list of impaired metabolic pathways differentiating sepsis requiring care in pediatric intensive care from sepsis cared for in the emergency department.

Metabolic pathway	p-value	Impact	Total	Hits
Pyruvate metabolism	3.7 × 10^−22^	0.1	32	Acetate
Alanine, aspartate and glutamate metabolism	3.6 × 10^−12^	0.06	24	Alanine
Sulfur metabolism	3.7 × 10^−22^	0.03	18	Acetate
Taurine and hypotaurine metabolism	2.2 × 10^−37^	0.03	20	Acetate, alanine
Fructose and mannose metabolism	2.2 × 10^−4^	0.03	48	Mannose
Selenoamino acid metabolism	2.2 × 10^−37^	0.003	22	Acetate, alanine

The p-value was calculated during the pathway enrichment analysis and was adjusted by Holm-Bonferroni method. The pathway Impact value was calculated from the pathway topology analysis as the sum of the importance measures of the matched metabolites normalized by the sum of the importance measures of all metabolites in each pathway. The Total is the total number of compounds in the pathway and the Hits show the actually matched metabolites detected as potentially important compounds in our study. The analysis was performed using the MetaboAnalyst 3.0 software^22^.

The elevated level of dimethylamine may indicate impairment in renal function as well as an excessive production of asymmetric dimethylarginine (ADMA) in sepsis^28,29^. Dimethylamine is created through the metabolism of ADMA which is an important regulator of the L-arginine/nitric oxide (NO) pathway^30^. ADMA acts as an endogenous inhibitor of NO synthase and is responsible for reduced NO availability, development of oxidative stress and dysfunction of the immune system response^31^. The increased concentrations of 3-methyl-2-oxovalerate and 3-hydroxyisovalerate point to organic acidemia, ketosis and disruption in branched-chain amino acid (BCAA) metabolism^32,33^. 3-Methyl-2-oxovalerate is a product of isoleucine degradation while 3-hydroxyisovalerate is created during leucine metabolism. Enhanced amino acid catabolism is a common event during the progression of sepsis as the body utilizes energy by increased protein breakdown and oxidation of BCAAs^34^. A high level of mannose suggests a reduction in aerobic glycolysis and the existence of a carbohydrate metabolic disorder in hepatic glycogen catabolism^35,36^. Alanine is an important energy source as it is a precursor of glucose in gluconeogenesis while acetate is one of the major components in TCA cycle and pyruvate metabolism^25^. Changes in the concentrations of mannose, alanine and acetate indicate altered requirements for energy in the PICU-sepsis patients when compared to the PED-sepsis cohort and probably provide a relative indicator of disease severity. A drop of the O-acetylcholine level suggests disruption in glycerophospholipid metabolism^25^. Moreover, O-acetylcholine is an important neurotransmitter which modifies the systemic inflammatory response^37^. These metabolic changes are similar to those in adults with severe sepsis, where increased amino acid catabolism, ketosis, altered TCA cycle and glucose metabolism, increased ADMA, and altered glycerophospholipid metabolism are reported^27,38–40^. Altogether, these results identify particular early metabolic changes induced by severe sepsis (Table 6 and Supplementary Fig. 1) that could become important indicators for PED staff to initiate appropriate and rapid treatment, and optimize patient stratification.

Another important finding in our study is that the supervised statistical analysis performed on the inflammatory protein-mediator profiling dataset was less able to clearly distinguish between PICU-sepsis patients and PED-sepsis patients in children aged 1–23 months compared to the metabolic profiling (AUC 0.84 ± 0.04 versus 0.90 ± 0.03). This is similar to our previous finding in older children (AUC 0.88 ± 0.03 versus 0.96 ± 0.01)^12^. In both age cohorts the combined metabolic and protein-mediator profiling did not seem to improve prediction significantly from the metabolic model alone. The response to sepsis cannot be easily classified as pro- or anti-inflammatory, as many regulatory processes are activated in exceedingly complex ways. From the innumerable genomic, transcriptomic, and proteomic (including inflammatory protein-mediators) interacting cascades occurring, a metabolic footprint of the organism’s metabolism may be found. Thus, it is possible that inflammatory protein-mediator data is redundant as these mediators act, in the end, to alter the metabolome of the organism. Interestingly, when we compared the metabolite/inflammatory protein-mediator patterns obtained for the OPLS-DA models PICU-sepsis versus PED-sepsis for infants and older children, only seven metabolites, and none of the protein-mediators were common for both pediatric age groups (Supplementary Tables S4, S5, and Fig. S1). This may reflect the gradual maturation of the immune system during early infancy, with differing responses to infection from older children^41^. Alternatively, and more in keeping with our hypothesis about the power of metabolomics, the different specific protein-mediators and metabolites identified in the two age cohorts may simply reflect differences in timing of presentation during the cascades occurring, while the common metabolites reflect the end-result of those cascades.

Limitations of this study should be recognized. First, the study included a modest cohort of 122 patients age 2–23 months and 175 children age 2–17 years from two centers in Alberta, with a consent rate of 78%. Although this study is the largest involving metabolomics in children with sepsis, is multicenter involving the only two PICUs in the province serving a population of over 4 million, and had a high consent rate, further prospective validation in a larger multicenter cohort would strengthen the findings. Second, our internal validation methodology of training and test data sets also requires prospective validation in an independent cohort. Third, the cohort definitions relied on the clinical triage decision and not a more objective method. For example, we did not require measurements of organ dysfunction in the PED-sepsis cohort, and thus cannot determine how a more objective organ dysfunction score compares to or adds information to the biopattern classifications. It is likely that the PED-sepsis cohort would have had lower PELOD scores than the PICU-sepsis cohort. However, the decision of whether to measure these laboratory indices of organ dysfunction is itself subjective (often based on clinician level of worry about triage to a PICU), and our aim was to determine an objective biopattern that tracks these real-world decisions. Moreover, if not measured these indices are assumed to be normal in calculating the scores. Since the goal was to track the decision to triage to PICU, comparing these scores between PICU- and PED-sepsis patients would have involved circular reasoning. Similarly, we did not use an ED serious infection prediction rule or Pediatric Early Warning Score (PEWS) because most of these scores include, in addition to vital signs, subjective descriptions of the level of consciousness, capillary refilling, work of breathing, and clinician ‘worry’, overall ‘concern’, or ‘gut feeling’ about clinical status^42–46^. This may be why evaluations of the existing serious infection prediction rules and PEWS have concluded that they have inadequate discriminant ability for filling the ‘diagnostic gap’ (i.e., predicting PICU admission or serious infections in the ED)^42–46^. We found that the clinical decisions and the discovered biopatterns differentiated a PICU cohort that had high need for ventilation and vasoactive infusions and a prolonged PICU length of stay, from a PED cohort that did not require the specialized and costly care of a PICU. Differentiating these cohorts may be particularly useful in hospitals without specialized PEDs and PICUs, which include most hospitals where children present for care. Fourth, the exact timing of onset of sepsis in patients is unclear, and the duration prior to having blood drawn was likely different between PED-sepsis (drawn with initial labwork done in the PED) and PICU-sepsis cohorts (done once in PICU for 96%). It is possible that the PICU-sepsis cohort biopatterns reflect illness management in the PICU (e.g., ventilation, vasoactive medications, and other resuscitation), rather than predicting these needs. An ideal study design would have been to have the blood drawn from all patients in the PED, prior to the triage decision being made. Our pragmatic deferred consent model in the PICU-sepsis cohort that ensured the blood was drawn on the day of eligibility, within 24 hours of presentation, may mitigate this concern.

In conclusion, in children age 1–23 months, metabolic and protein-mediator profiling early in presentation can differentiate infants with sepsis requiring care in a PICU from infants with and without sepsis safely cared for outside of a PICU. In children age 1 month-17 years, a biopattern for sepsis triage consisting of 7 metabolites found early in patient presentation was internally validated using development and test sets of children in our cohorts. This biopattern mostly reflects altered energy metabolism in more severely affected children with sepsis. This discovery systems-biology based approach identified a limited number of metabolites-of-interest that may realistically lead to the development of a point-of-care sepsis triage decision aid, which may be particularly useful in ED without pediatric expertise. This finding requires prospective validation in an independent cohort, ideally with comparison to and/or addition to clinical prediction scores..

## Electronic supplementary material

Supplementary Information

## Data Availability

The datasets generated and analyzed during the current study are available from the corresponding author on reasonable request.
