# Supplementary material for: Biomarker Phenotype for Early Diagnosis and Triage of Sepsis to the Pediatric Intensive Care Unit
Source: Sci Rep. 2018 Nov 9;8:16606. doi: 10.1038/s41598-018-35000-7 (PMC6226431; doi:10.1038/s41598-018-35000-7)
Supplement: Supplementary file 1 — Supplementary Information [file 41598_2018_35000_MOESM1_ESM.docx]

**Supplementary Information**

**Biomarker Phenotype for Early Diagnosis and Triage of Sepsis to the Pediatric Intensive Care Unit**

Authors: Beata Mickiewicz^1^, Graham C. Thompson^2^, Jaime Blackwood^2^, Craig N. Jenne^3^, Brent W. Winston^4^, Hans J. Vogel^1^, Ari R. Joffe^5*^

**Author Affiliations:** ^1^ Bio-NMR-Centre, Department of Biological Sciences, Faculty of Science, University of Calgary, Calgary, AB, Canada; ^2^ Department of Pediatrics, University of Calgary, Calgary, AB, Canada; ^3^ Department of Microbiology, Immunology and Infectious Diseases, Cumming School of Medicine, University of Calgary, Calgary, AB, Canada; ^4^ Department of Critical Care Medicine, Department of Medicine, Department of Biochemistry and Molecular Biology, Cumming School of Medicine, University of Calgary, Calgary, AB, Canada; ^5^ Division of Pediatric Critical Care Medicine, Department of Pediatrics, University of Alberta, Edmonton, AB, Canada

***Corresponding Author:** Ari R. Joffe; 4-546 Edmonton Clinic Health Academy; 11405 87 Ave; Edmonton, Alberta, Canada, T6G1C9, [ari.joffe@albertahealthservices.ca], 780-248-5435.

**Supplemental Methods:**

***^1^H Nuclear magnetic resonance (NMR) spectroscopy***

Sample preparation: Serum samples (V=150μl) were thawed and filtered twice using 3kDa NanoSep microcentrifuge filters (VWR International, Edmonton, AB, Canada). Next, the filtrate was brought to 400μl by adding 80μl of a sodium phosphate buffer solution (0.5M NaH_2_PO_4_ containing internal standard: 2.5mM 2,2-dimethyl-2-silapentane-5-sulfonate, DSS; final concentration of DSS was 0.5mM, pH=7.0±0.04), 10μl of sodium azide (1M NaN_3_) and D_2_O.

NMR spectral acquisition: High resolution one-dimensional ^1^H NMR spectra were obtained on a 600 MHz Bruker Ultrashield Plus NMR spectrometer (Bruker BioSpin Ltd., Canada) applying a standard Bruker 1D spectroscopy presaturation pulse sequence (noesypr1d) with optimal water suppression and a mixing time of 100ms (1,2). The spectra were manually corrected (phasing, baseline correction, referencing to the DSS peak at 0.0 ppm) and analyzed using a quantitative profiling approach(2) (Chenomx NMR Suite 7.5 software; Chenomx Inc., Edmonton, AB, Canada). The concentration of DSS was used as the internal reference to determine the concentrations of detected metabolites. Additionally, the chemical shift assignments were confirmed and verified with the Human Metabolome Database (version 3.0) (3) and 2D NMR spectra (total correlation spectra and ^1^H, ^13^C heteronuclear single quantum coherence spectra). All samples and ^1^H NMR spectra were randomized prior to analysis to avoid progressive bias.

***Statistical analysis***

All metabolites or protein-mediators with more than 50% missing values were left out from the statistical analysis i.e. metabolite peaks that could not be distinguished from noise in the NMR spectra; protein-mediators values below the limit of detection, showing “out of range” values, or with the coefficient of variance between two replicates more than 20%*.* The data were pre-processed: median fold change normalization, logarithmic transformation, centering and unit variance scaling (4). The metabolic and inflammatory protein-mediator data were analyzed separately and in a combined fashion (integrated metabolic and protein-mediator dataset).

Multivariate statistical analysis was performed in the SIMCA-P+ 12.0.1 software (Umetrics, Sweden) and consisted of unsupervised principal component analysis (PCA) and supervised method of orthogonal partial least squares discriminant analysis (OPLS-DA). The PCA was applied to summarize the source of variation in each dataset and to show outlying samples, i.e. samples that are situated outside of the 95% confidence interval of the Hotelling’s T-squared distribution in the score scatter plots (5). The OPLS-DA method was carried out to improve model transparency and its interpretability. The OPLS-DA models were based on metabolites with Variable Influence on Projection (VIP) values larger than 1 (5,6). When the groups of samples in the OPLS-DA models are of greatly unequal size, it becomes difficult to interpret the results and it is nearly impossible to judge fairly the statistical significance of the discriminant function (5,7). Therefore, the OPLS-DA models, which included imbalanced number of samples (ratio between group sizes not more than 2:1) were constructed based on age-matched samples. To validate the statistical significance of each OPLS-DA model the following parameters were calculated based on sevenfold cross-validation (CV) (8): R2Y (the percentage of variation explained by the model), Q2 (the predictive ability of the model) and CV-ANOVA (Cross-Validated Analysis Of Variance) p-value (5,9). The potentially important metabolites and protein-mediators were based on the OPLS-DA regression coefficients (p<0.05; jackknife technique) (10).

In order to confirm the most significant metabolites and inflammatory protein-mediators, which distinguish between PICU-sepsis and PED-sepsis for pediatric patients, the results obtained in infants were compared to our previously published results for metabolite and inflammatory protein-mediator profiling for an older pediatric patient cohort (2-17 year old children) (11). The metabolites and protein-mediators common for both patient cohorts were considered as potential biomarkers (*biopattern*). Based on these potential biomarkers an alternative OPLS-DA model was created: PICU-sepsis versus PED-sepsis for infants and older children respectively. The receiver operating characteristic (ROC) analysis (12) for each single potential biomarker as well for the multivariate *biopattern* was performed using the MetaboAnalyst 3.0 software (13). The multivariate exploratory ROC curves were generated by Monte-Carlo cross validation (MCCV=3) using the build-in PLS-DA algorithm for feature selection and performance evaluation procedure (13). Additionally, for each OPLS-DA model an area under the ROC curve (AUROC) was calculated (Metz ROC Sofware, The University of Chicago) where specificity, sensitivity and accuracy were based on the Y-predCV values (sample class prediction during the cross-validation).

To further validate a supervised model PICU-sepsis versus PED-sepsis based on potential biomarkers, the OPLS-DA model which consisted of 2/3 of all available pediatric samples (combined PICU-sepsis and PED-sepsis samples from younger and older pediatric cohort) was used as a training set, while the remaining samples comprised a test set. The model was validated 3 times and for each training set the samples were randomly selected. During model construction, the training PED-sepsis samples were assigned a response value of zero (0.0), while the PICU-sepsis samples were given a response of one (1.0). During validation analysis if the predicted response value was more than 0.5, the sample would be predicted as a PICU-sepsis sample. Otherwise the unknown test sample was assigned to the PED-sepsis cohort. The data obtained for the test set i.e. Y-predPS values (predicted response values for the test set samples) were used to calculate the sensitivity, specificity and evaluate the accuracy of the model’s prediction.

**References:**

1. Nicholson, J.K., Foxall, P.J., Spraul, M., Farrant, R.D. & Lindon, J.C. 750 MHz 1H and 1H-13C NMR spectroscopy of human blood plasma. *Anal Chem*. **67**, 793-811 (1995).

2. Weljie, A.M., Newton, J., Mercier, P., Carlson. E. & Slupsky, C.M. Targeted profiling: quantitative analysis of 1H NMR metabolomics data. *Anal Chem*. **78**, 4430-4442 (2006).

3. Wishart, D.S. *et al*. HMDB 3.0--The Human Metabolome Database in 2013. *Nucleic Acids Res.* **41**, D801-7 (2013).

4. van den Berg, R.A., Hoefsloot, H.C., Westerhuis, J.A., Smilde, A.K. & van der Werf, M.J. Centering, scaling, and transformations: improving the biological information content of metabolomics data. *BMC Genomics*. **7**, 142 (2006).

5. Eriksson, L. *et al*. Multi- and Megavariate Data Analysis Part I: Basic Principles and Applications (ed. Eriksson, L. *et al*.) (Umeå, Sweden: Umetrics AB, 2006).

6. Galindo-Prieto, B., Eriksson, L. & Trygg, J. Variable influence on projection (VIP) for orthogonal projections to latent structures (OPLS). *J Chemom*. **28**, 623-632 (2014).

7. Bylesjo, M. *et al*. OPLS discriminant analysis: combining the strengths of PLS-DA and SIMCA classification. *J Chemom*. **20**, 341-351 (2006).

8. Picard, R.R. & Cook, D.R. Cross-Validation of Regression Models. *J Amer Stat Assoc.* **79**, 575-583 (1984).

9. Eriksson, L., Trygg, J. & Wold, S. CV-ANOVA for significance testing of PLS and OPLS models. *J Chemom*. **22**, 594-600 (2008).

10. Trygg, J., Holmes, E. & Lundstedt, T. Chemometrics in metabonomics. *J Proteome Res.* **6**, 469-479 (2007).

11. Mickiewicz, B. *et al*. Development of metabolic and inflammatory mediator biomarker phenotyping for early diagnosis and triage of pediatric sepsis. *Crit Care.* **19**, 320 (2015).

12. Metz, C.E. Basic principles of ROC analysis. *Semin Nucl Med.* **8**, 283-298 (1978).

**Supplemental Tables.**

**Table S1.** List of detected metabolites with the chemical shifts used to make metabolite assignments in ^1^H NMR spectra of serum samples.

| **Compound Name** | **Chemical Shifts** [± 0.025 ppm] |
| --- | --- |
| 2-Aminobutyrate | 0.97; 1.89; 3.71 |
| 2-Hydroxybutyrate | 0.89; 1.64; 1.73; 3.99 |
| 2-Hydroxyisovalerate | 0.82; 0.95; 2.01; 3.84 |
| 2-Oxoglutarate | 2.43; 3.00 |
| 2-Oxoisocaproate | 0.92; 2.08; 2.60 |
| 3-Hydroxybutyrate | 1.19; 2.30; 2.39; 4.14 |
| 3-Hydroxyisovalerate | 1.26; 2.35 |
| 3-Methyl-2-oxovalerate | 0.88; 1.11; 1.45; 1.69; 2.92 |
| Acetaminophen | 2.15; 6.90; 7.24 |
| Acetate | 1.91 |
| Acetoacetate | 2.27; 3.44 |
| Acetone | 2.22 |
| Alanine | 1.47; 3.78 |
| Arginine | 1.64; 1.72; 1.89; 1.92; 3.24; 3.76; 6.67; 7.23 |
| Asparagine | 2.85; 2.94; 4.00; 6.91; 7.62 |
| Aspartate | 2.66; 2.80; 3.89 |
| Betaine | 3.25; 3.89 |
| Carnitine | 2.41; 2.45; 3.21; 3.40; 3.43; 4.56 |
| Choline | 3.19; 3.51; 4.06 |
| Citrate | 2.53; 2.69 |
| Creatine | 3.03; 3.92 |
| Creatine phosphate | 3.03; 3.94 |
| Creatinine | 3.03; 4.05 |
| DSS (internal standard) | 0.00; 0.62; 1.75; 2.91 |
| Dimethyl sulfone | 3.14 |
| Dimethylamine | 2.72 |
| Ethanol | 1.17; 3.65 |
| Formate | 8.44 |
| Glucose | 3.24; 3.39; 3.40; 3.46; 3.48; 3.53; 3.70; 3.72; 3.76; 3.82; 3.84; 3.89; 4.64; 5.23 |
| Glutamate | 2.04; 2.12; 2.33; 2.36; 3.75 |
| Glutamine | 2.11; 2.14; 2.43; 2.46; 3.77; 6.87; 7.59 |
| Glycerol | 3.55; 3.65; 3.78 |
| Glycine | 3.55 |
| Histidine | 3.14; 3.24; 3.98; 7.09; 7.88 |
| Hypoxanthine | 8.18; 8.20 |
| Isobutyrate | 1.05; 2.38 |
| Isoleucine | 0.93; 1.00; 1.25; 1.46; 1.97; 3.66 |
| Isopropanol | 1.16; 4.01 |
| Lactate | 1.32; 4.11 |
| Leucine | 0.94; 0.95; 1.67; 1.70; 1.73; 3.72 |
| Lysine | 1.43; 1.50; 1.72; 1.88; 1.91; 3.02; 3.75 |
| Malonate | 3.11 |
| Mannose | 3.37; 3.57; 3.65; 3.65; 3.73; 3.76; 3.81; 3.84; 3.87; 3.90; 3.93; 3.94; 4.89; 5.17 |
| Methanol | 3.35 |
| Methionine | 2.11; 2.13; 2.19; 2.63; 3.85 |
| myo-Inositol | 3.27; 3.53; 3.61; 4.06 |
| O-Acetylcarnitine | 2.13; 2.50; 2.63; 3.18; 3.60; 3.84; 5.59 |
| O-Acetylcholine | 2.14; 3.20; 3.72; 4.55 |
| Ornithine | 1.74; 1.82; 1.93; 3.05; 3.78 |
| Phenylalanine | 3.11; 3.27; 3.99; 7.32; 7.37; 7.42 |
| Proline | 1.98; 2.02; 2.34; 3.33; 3.41; 4.12 |
| Propylene glycol | 1.13; 3.43; 3.54; 3.87 |
| Pyruvate | 2.36 |
| Serine | 3.84; 3.94; 3.98 |
| Taurine | 3.25; 3.42 |
| Threonine | 1.32; 3.58; 4.25 |
| Trimethylamine N-oxide | 3.25 |
| Tyrosine | 3.04; 3.19; 3.93; 6.89; 7.18 |
| Urea | 5.77 |
| Valine | 0.98; 1.03; 2.26; 3.60 |

**Table S2.** List of protein-mediators recognized and quantified in plasma samples.

| IL-1β  IL-1Ra  IL-2  IL-4  IL-5  IL-6  IL-7  IL-8  IL-9  IL-10  IL-12(p70)  IL-13  IL-15  IL-17  Eotaxin  FGF basic  G-CSF  GM-CSF  IFN-γ  IP-10  MCP-1(MCAF)  MIP-1α  PDGF-ββ  MIP-1β  RANTES  TNF-α  VEGF  IL-1α  IL-2Rα | IL-3  IL-12p40  IL-16  IL-18  CTACK  GROa  HGF  IFN-α2  LIF  MCP-3  M-CSF  MIF  MIG  β-NGF  SCF  SCGF-β  SDF-1α  TNF-β  TRAIL  PCT  Ferritin  tPA  A2M  SAP  Fibrinogen  SAA  Haptoglobin  CRP |
| --- | --- |

**Table S3.** Comparison of statistical measures calculated for the supervised OPLS-DA models with and without excluding outliers in infants age 1-23 months.

| **Data** | **Outliers** | **OPLS-DA** | | |
| --- | --- | --- | --- | --- |
|  |  | **PICU-sepsis versus PED-sepsis** | **PED-sepsis versus PED-controls** | **PICU sepsis versus PED-controls** |
| Metabolic profiling dataset | Outliers included | R2Y=0.47  Q2=0.43 | R2Y=0.72  Q2=0.61 | R2Y=0.91  Q2=0.83 |
|  | Outliers excluded (see manuscript) | R2Y=0.48  Q2=0.44 | R2Y=0.72  Q2=0.61 | R2Y=0.93  Q2=0.88 |
| Protein-mediator profiling dataset | Outliers included | R2Y=0.37  Q2=0.30 | R2Y=0.81  Q2=0.74 | R2Y=0.81  Q2=0.63 |
|  | Outliers excluded (see manuscript) | R2Y=0.37  Q2=0.32 | R2Y=0.77  Q2=0.75 | R2Y=0.86  Q2=0.67 |
| Combined metabolic and protein-mediator profiling dataset | Outliers included | R2Y=0.43  Q2=0.39 | R2Y=0.80  Q2=0.70 | R2Y=0.89  Q2=0.84 |
|  | Outliers excluded (see manuscript) | R2Y=0.45  Q2=0.39 | R2Y=0.80  Q2=0.70 | R2Y=0.94  Q2=0.90 |

**Table S4.** List of potentially important metabolites and protein mediators for the orthogonal partial least squares discriminant analysis model differentiating sepsis requiring care in pediatric intensive care from sepsis cared for in the emergency department in infants age 1-23 months

| **Concentration increased ↑** | **Concentration decreased ↓** |
| --- | --- |
| Procalcitonin  Dimethylamine  Mannose  Ferritin  Interleukin-8  β-Nerve growth factor  Hepatocyte growth factor  Acetoacetate  Creatinine  3-Methyl-2-oxovalerate  Stem cell growth factor-β  Glucose  Interleukin-2 receptor α  Granulocyte-colony stimulating factor  3-Hydroxyisovalerate  Interleukin-6  Tissue plasminogen activator  Stromal cell-derived factor-1β  Acetone | Alanine  Valine  Carnitine  O-Acetylcholine  Leucine  Isoleucine  Ornithine  RANTES  Acetate  Proline  Interleukin-1β  Fibroblast growth factor  Lactate  Creatine  Tyrosine  Platelet-derived growth factor-ββ  2-Aminobutyrate |

**Table S5.** List of potentially important metabolites and protein-mediators (OPLS-DA regression coefficients, p<0.05) based on the combined metabolic/protein-mediator profiling dataset for the OPLS-DA model in children age 2-17 years: PICU-sepsis versus PED-sepsis (1).
**↑**: metabolite/protein-mediator concentration increased, **↓**: metabolite/protein-mediator concentration decreased in PICU-sepsis samples as compared to PED-sepsis samples.

| PICU-sepsis versus PED-sepsis |
| --- |
| Mannose **↑**  Propylene glycol **↑**  Dimethylamine **↑**  2-Hydroxyisovalerate **↑**  3-Methyl-2-oxovalerate **↑**  2-Oxoisocaproate **↑**  2-Hydorxybutyrate **↑**  3-Hydroxyisovalerate **↑**  Choline **↓**  Alanine **↓**  Dimethyl sulfone **↓**  O-Acetylcholine **↓**  α-2-Macroglobulin **↓**  Taurine **↓**  Serum amyloid A **↓**  TRAIL **↓**  Acetate **↓** |

1. Mickiewicz, B. *et al*. Development of metabolic and inflammatory mediator biomarker phenotyping for early diagnosis and triage of pediatric sepsis. *Crit Care*. **19**, 320 (2015).

**Supplemental Figure S1.**  The OPLS-DA regression coefficient plots based on the combined metabolic/protein-mediator profiling dataset for the OPLS-DA model PICU-sepsis versus PED-sepsis in (a) infants age 1-23 months and (b) children age 2-17 years: (1). Positive values of coefficients (the upper part of the diagrams) indicate increased metabolite/protein-mediator concentrations in PICU-sepsis samples (fold change>1) while negative values (the lower part of diagrams) present a decrease in metabolite/protein-mediator concentrations, as compared to PED-sepsis samples (fold change<1). Only significant metabolite/protein-mediator are shown (p<0.05; jackknife technique). Common metabolites in both cohorts are marked in red.


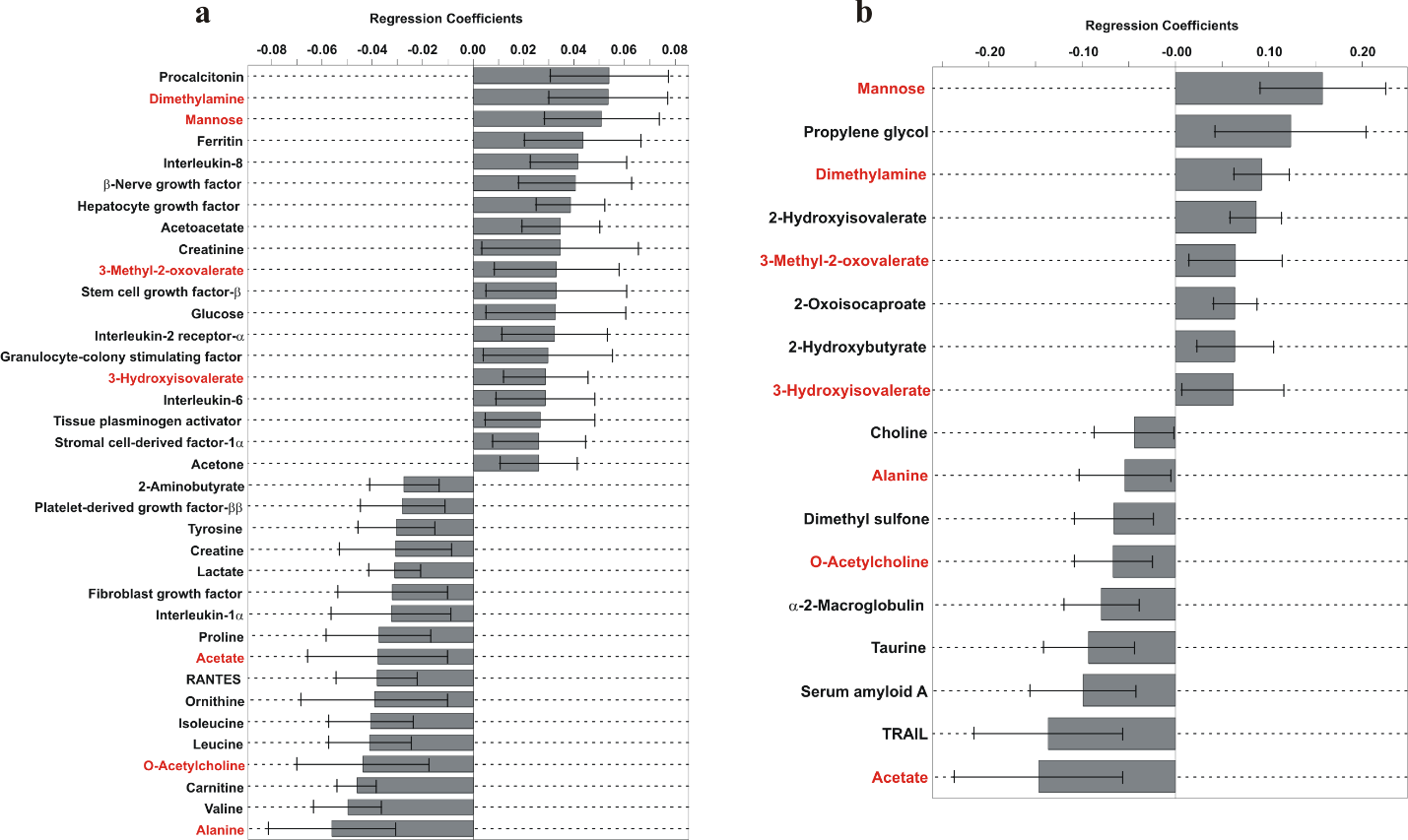


1. Mickiewicz B, Thompson GC, Blackwood J, et al. Development of metabolic and inflammatory mediator biomarker phenotyping for early diagnosis and triage of pediatric sepsis. Crit Care 2015;19:320.
